# Cytotoxic targeting of F9 teratocarcinoma tumours with anti-ED-B fibronectin scFv antibody modified liposomes

**DOI:** 10.1038/sj.bjc.6600423

**Published:** 2002-07-15

**Authors:** C Marty, B Odermatt, H Schott, D Neri, K Ballmer-Hofer, R Klemenz, R A Schwendener

**Affiliations:** Department of Pathology, University Hospital Zurich, Schmelzbergstrasse 12, CH-8091 Zurich, Switzerland; Institute of Organic Chemistry, Eberhard-Karls University, Auf der Morgenstelle 18, D-72076 Tuebingen, Germany; Department of Applied Biosciences, Swiss Federal Institute of Technology, Winterthurerstrasse 190, CH-8057, Zurich, Switzerland; Institute of Medical Radiobiology of the University of Zurich and Paul Scherrer Institute, CH-5232 Villigen-PSI, Switzerland

**Keywords:** fibronectin, tumour targeting, immunoliposomes, scFv antibody

## Abstract

We prepared small unilamellar liposomes derivatised with single chain antibody fragments specific for the ED-B domain of B-fibronectin. This extracellular matrix associated protein is expressed around newly forming blood vessels in the vicinity of many types of tumours. The single chain antibody fragments were functionalised by introduction of C-terminal cysteines and linked to liposomes via maleimide groups located at the terminal ends of poly(ethylene glycol) modified phospholipids. The properties of these anti-ED-B single chain antibody fragments-liposomes were analysed *in vitro* on ED-B fibronectin expressing Caco-2 cells and *in vivo* by studying their biodistribution and their therapeutic potential in mice bearing subcutanous F9 teratocarcinoma tumours. Radioactively labelled (^114m^Indium) single chain antibody fragments-liposomes accumulated in the tumours at 2–3-fold higher concentrations during the first 2 h after i.v. injection compared to unmodified liposomes. After 6–24 h both liposome types were found in similar amounts (8–10% injected dose g^−1^) in the tumours. Animals treated i.v. with single chain antibody fragments-liposomes containing the new cytotoxic agent 2′-deoxy-5-fluorouridylyl-N^4^-octadecyl-1-β-D-arabinofuranosylcytosine (30 mg kg^-1^ per dose, five times every 24 h) showed a reduction of tumour growth by 62–90% determined on days 5 and 8, respectively, compared to animals receiving control liposomes. Histological analysis revealed a marked reduction of F9 tumour cells and excessive deposition of fibronectin in the extracellular matrix after treatment with single chain antibody fragments-2-dioxy-5-fluorouridylyl-N^4^-octadecyl-1-β-D-arabinofuranosylcytosine-liposomes. Single chain antibody fragments-liposomes targeted to ED-B fibronectin positive tumours therefore represent a promising and versatile novel drug delivery system for the treatment of tumours.

*British Journal of Cancer* (2002) **87**, 106–112. doi:10.1038/sj.bjc.6600423 www.bjcancer.com

© 2002 Cancer Research UK

## 

Specific targeting of cytotoxic compounds to the tumour vasculature and the surrounding stroma of growing tumours would offer various advantages compared to conventional systemic chemotherapy. Fibronectin (Fn), in particular the B-Fn isoform with the ED-B oncofetal domain represents such a target protein that is expressed in and around neoplastic blood vessels during tumour growth and angiogenesis (Potts and Campbell, 1996; Ruoslahti, 1999). ED-B Fn is produced by a variety of tumour and endothelial cell lines and it was also found in a large number of breast, prostate and colorectal carcinoma tissue samples of mixed histotypes (Zardi *et al*, 1987; Kosmehl *et al*, 1996; Albrecht *et al*, 1999; Midulla *et al*, 2000). Therefore, the ED-B isoform of Fn represents a promising marker in growing solid tumours. Fn is an extracellular adhesion glycoprotein that mediates interactions between cells and extracellular matrix components. Fn polymorphism originates from alternative splicing of the primary transcript of a single gene in three regions.

Carnemolla characterised a single chain Fv antibody fragment (scFv) binding to the human ED-B domain of the B-Fn isoform using phage display technology (Carnemolla *et al*, 1996). Recently, Neri and coworkers have prepared new anti-ED-B scFv with high binding affinity (Tarli *et al*, 1999; Viti *et al*, 1999) and a tissue factor-ED-B-scFv fusion protein that mediated infarction of solid tumours (Nilsson *et al*, 2001).

Liposomes are widely used as delivery systems with a broad spectrum of agents including chemotherapeutics, imaging agents, antigens, lipids and DNA (Mastrobattista *et al*, 1999). Long-circulating poly(ethylene) glycol (PEG) modified liposomes are used as carriers for a variety of drugs (Cabanes *et al*, 1999). Targeting of liposomes to sites of drug action is achieved by attachment of specific antibodies or antibody fragments to their surface (Schwendener *et al*, 1990; Hansen *et al*, 1995; de Kruif *et al*, 1996). In this work we modified liposomes containing NH_2_-substituted PEG-chains with the bifunctional reagent sulfosuccinimidyl-4-(N-maleimidomethyl)cyclohexane-1-carboxylate (sulfo-SMCC) for the introduction of maleimide, allowing stable covalent S-C-linkage with C-terminal cysteine modified anti-ED-B scFv (Marty *et al*, 2001).

Specific binding of the anti-ED-B-scFv-liposomes (scFv-liposomes) was demonstrated on the ED-B expressing colorectal carcinoma cell line Caco-2 (Pujuguet *et al*, 1996). The biodistribution of ^114m^In-labelled liposomes was compared in mice bearing F9 teratocarcinoma tumours (Neri *et al*, 1997). Antitumour activity of scFv-liposomes *vs* unmodified liposomes was shown by loading the liposomes with 2′-deoxy-5-fluorouridylyl-N^4^-octadecyl-1-β-D-arabinofuranosylcytosine (5-FdU-NOAC), a new amphiphilic compound with high cytotoxic activity (Cattaneo-Pangrazzi *et al*, 2000).

## MATERIALS AND METHODS

### Chemicals

Soy phosphatidylcholine (SPC) was obtained from L Meyer (Hamburg, Germany). Cholesterol (CHOL) was purchased from Fluka (Buchs, Switzerland). Methoxy-poly(ethylene glycol)-phosphatidylethanolamine (PE-PEG-OMet) was from Sygena (Liestal, Switzerland). Amino-poly(ethylene glycol)-phosphatidylethanolamine (PE-PEG-NH_2_) was from Shearwater Polymers, Inc. (Enschede, The Netherlands). 3,3′-dioctadecyloxacarbocyanine perchlorate (DiO) and maleimide-BODIPY (4,4-difluoro-1,3,5,7-tetramethyl-4-bora-3a,4a-diaza-s-indacene-8-propionic acid) were from Molecular Probes (Leiden, The Netherlands). 5-FdU-NOAC was synthesised as described by Cattaneo-Pangrazzi *et al* (2000). ^114m^InCl_3_ was from NEN Life Science Products (Boston, MA, USA) and [5-^3^H]-N^4^-octadecyl-1-β-D-arabinofuranosylcytosine (5-^3^[H]-NOAC) was custom labelled by Amersham Int. (Amersham, UK). Sulfo-SMCC was from Pierce (Lausanne, Switzerland). Dulbecco's modified Eagles medium (DMEM), foetal bovine serum and all medium supplements were from Gibco BRL (Basel, Switzerland). All buffer salts and other chemicals were of analytical grade and obtained from Fluka or Sigma (Buchs, Switzerland).

### Cell lines and animals

The human colon carcinoma cell line Caco-2 (H Wunderli-Allenspach, Swiss Federal Institute of Technology, Zurich, Switzerland) was maintained in DMEM plus 10% heat-inactivated foetal bovine serum, 1% L-glutamine, 1% nonessential amino acids, 1 mM sodium pyruvate, 100 U ml^−1^ penicillin and 100 μg ml^−1^ streptomycin. The human colon carcinoma cell line Co-115 (B Sordat, Swiss Institute for Cancer Research, ISREC, Lausanne, Switzerland) and the murine F9 teratocarcinoma cell line (Neri *et al*, 1997) were maintained in DMEM as described above but without nonessential amino acids and pyruvate. Female nude mice (CD-1<R>)-nu/nu were obtained from Charles River Wiga (Sulzfeld, Germany) and kept in standard housing and normal diet at the animal facility of the University Hospital Zurich. All animal studies were performed under a license (Nr. 141/98) issued to RA Schwendener by the Veterinary Department of the Canton Zurich. The ethical guidelines that were followed meet the standards required by the UKCCCR guidelines (Workman *et al*, 1998).

### Liposome preparation, modification and labelling

The liposomes were composed of SPC : CHOL : PE-PEG-OMet (unmodified liposomes) or PE-PEG-NH_2_ (scFv modified liposomes) at a molar ratio of 1 : 0.2 : 0.07. For the *in vitro* binding experiments liposomes were labelled with the lipophilic dye DiO (0.004 mol parts referred to SPC). Maleimide-BODIPY was used for the determination of the modification efficiency of NH_2_-PEG-PE. For therapeutic studies 5-FdU-NOAC (0.075 mol parts referred to SPC) and 5-^3^[H]-NOAC as trace label for the drug were added to the lipid mixture.

All small unilamellar liposomes (SUV) were prepared by sequential filter extrusion of multilamellar liposomal preparations in phosphate buffer (PB, 67 mM, pH 7.4) through Nuclepore™ membranes (Sterico, Dietikon, Switzerland) of 0.2 and 0.1 μm pore diameter with a Lipex™ extruder (Lipex Biomembranes Inc., Vancouver, Canada). Size and stability of the liposomes were analysed with a particle sizer (Nicomp Model 370, Santa Barbara, USA). For the biodistribution experiments liposomes containing A23187 ionophore (0.001 mol parts referred to SPC) and nitrilotriacetic acid (1 mM) were labelled with ^114m^InCl_3_ as described by Proffitt *et al* (1983). Briefly, ^114m^InCl_3_ (7×10^6^ c.p.m. per 100 μl liposomes, 80 mg SPC ml^−1^) was incubated with unmodified control liposomes for 30 min at 60°C and with scFv-liposomes for 2 h at RT. Free ^114m^In^3+^ was removed by size exclusion chromatography on a Sephadex G-50 column (Pharmacia, Uppsala, Sweden). ^114m^In entrapment was determined with a gamma counter (Cobra, Packard Instruments, Downers Grove, IL, USA).

### Production and characterisation of anti-ED-B scFv

The scFv were constructed, produced, purified and characterised as described elsewhere (Marty *et al*, 2001). Briefly, the sequence of anti-ED-B scFv (CGS-1) was modified by PCR using pDN351 as a template (Neri *et al*, 1997) to functionalise the antibody by introduction of additional cysteines at the C-terminal end, cloned into the pPICZαA vector and transformed into *Pichia pastoris*. The protein was produced by fermentation at medium-scale quantities and purified in its dimeric form over an anion exchange column.

### Preparation of scFv-liposomes

Liposomes containing 0.07 mol parts PE-PEG-NH_2_ referred to SPC in PB were incubated with crystalline sulfo-SMCC at a molar ratio of PEG-amino to maleimide groups of 1 : 5 for 30 min at 30°C. Excess sulfo-SMCC was removed on a Biogel P6 column (BioRad, Glattbrugg, Switzerland) in HBSE buffer (10 mM HEPES, 150 mM NaCl, 9.1 mM EDTA, pH 7.5). After reduction of the scFv dimer (0.5 mg ml^−1^) in HBSE with a 2 mM final concentration of tributylphosphine (TBP) for 4 h at 4°C sulfo-SMCC modified liposomes in HBSE were added and incubated for 20 h at 4°C under an argon atmosphere. Modified liposomes and non-reacted scFv were separated on a metrizamide gradient by ultracentrifugation (7 h, 85 000 **g**, 4°C, Schwendener *et al*, 1990). Liposome fractions were analysed by gel electrophoresis for their protein content.

### Binding of scFv-liposomes *in vitro*

Cover slips were placed into 12 well plates, coated with 100 μl well^−1^ rat tail collagen-I (10 mg ml^−1^) and incubated for 30 min at 37°C. Caco-2 and Co-115 cells (3×10^5^ cells well^−1^) were plated and cultured for 48 h in a humidified 5% CO_2_ atmosphere at 37°C. Washed cells were incubated for 30 min at 4°C with 100 μl DiO labelled liposomes. Washed cover slips were removed, treated with 10% glycerine in PB, placed on glass slides and analysed on a fluorescence microscope (Leica DLMB). As negative controls the cells were incubated with unmodified fluorescence labelled liposomes.

### Biodistribution of ^114m^In labelled scFv-liposomes in tumour bearing mice

**Figure 3:**
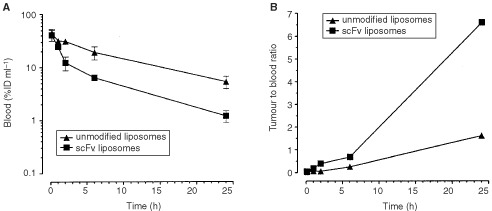
Blood distribution curves (**A**) and tumour to blood ratios (**B**) of unmodified liposomes and scFv-liposomes. Mice (three per group) bearing s.c. F9 tumours were injected i.v. with ^114m^In labelled liposomes. Results are expressed as % injected dose (%ID ml^−1^ ±s.e.m.) of radioactivity per millilitre blood or %ID g^−1^ ±s.e.m. tumour.

CD-1<R>-nu/nu mice received 10^7^ F9 cells per 50 μl PB s.c. on both sides of the back. As tumours had reached sizes of 0.5–1.0 cm in diameter, 200 μl ^114m^In labelled liposomes containing 5 mg SPC and 37.5 μg scFv in PB were injected i.v. Five minutes, 1, 2, 6 and 24 h later the animals were anaesthetised, and sacrificed by heart puncture and blood, heart, lung, liver, spleen, kidneys and tumours were removed, weighed and the radioactivity measured by gamma counting (Cobra, Packard Instruments). Blood correction factors were applied to all organ samples (Allen *et al*, 1991). Statistical parameters, the area under the curve (AUC) and half-lives of the blood distribution curves (Figure 3A

) were calculated with the GraphPad Prism graphical software.

### Therapeutic studies

F9 cells (10^7^ per 50 μl PB) were injected s.c. on the abdominal side of CD-1<R>-nu/nu mice. Mice (5–10 per group) were treated i.v. as the tumours had reached diameters of 6–8 mm with scFv-5-FdU-NOAC-liposomes, 5-FdU-NOAC-liposomes, scFv-liposomes, unmodified liposomes or scFv-dimers every 24 h for 5 days. Each dose (0.2 ml) contained 0.6 mg 5-FdU-NOAC (30 mg kg^−1^ per dose) or corresponding amounts of lipids or protein. Tumour growth was measured in a blinded fashion with a caliper every day and volumes calculated using the following equation: V=π ab^2^/6 (a=largest tumour diameter, b=perpendicular diameter). Tumour v w^−1^ values were converted into percent change from baseline using equation V_t_×100/V_0_ (V_t_=tumour volume at time t, V_0_=baseline volume). Mice were sacrificed 8 days after onset of treatment and tumours were dissected and weighed.

### Histology

Samples of F9 tumours were collected 72 h after one treatment (0.6 mg 5-FdU-NOAC) and histology was done as described by Roscic-Mrkic *et al* (2001). Sections were incubated with anti-ED-B Fn scFv L19 containing a FLAG tag (Viti *et al*, 1999), followed by a FITC-labelled mouse anti FLAG M2 monoclonal antibody (Sigma Chemical Co., St. Louis, MO, USA). FITC was detected by sequential incubation with a rabbit anti-FITC antibody (Dako, Glostrup, Denmark) and alkaline phosphatase (AP) and labelled donkey antibodies against rabbit IgGs (Jackson Laboratories). AP was visualised using naphthol AS-BI (6-bromo-2-hydroxy-3-naphtholic acid-2-methoxy anilide) phosphate and new fuchsin (Sigma). Sections were counterstained with hemalum.

## RESULTS

### Liposome preparation and scFv coupling

Total liposome numbers and the number of all lipophilic molecules per liposome were calculated from the mean diameter obtained from laser light scattering data and from liposome vesicle geometry parameters as described by Huang and Mason (1978). An average of 1670 accessible maleimide groups per liposome of 100 nm diameter prepared with 0.07 mol PE-PEG-NH_2_ were calculated. The efficiency of the modification with sulfo-SMCC was determined by fluorescence labelling of liposomes with maleimide-BODIPY resulting in 60% of maleimide modified PEG-NH_2_-groups (data not shown).

In the absence of reducing agents the anti-ED-B scFv constructs form dimers due to disulfide bridge formation (data not shown; Marty *et al*, 2001). Therefore, the disulfide bridges had to be cleaved prior to coupling to maleimide modified liposomes. In contrast to dithionite, that inactivated the binding properties of the scFv (data not shown), reduction of the dimers with 2 mM tributylphosphine for 4 h at 4°C produced active and stable monomers. Consequently, the scFv were coupled to the distal end of the PEG chain via the cysteine thiol groups in the presence of the reducing agent. Non-reacted scFv were separated from liposomes on a metrizamide gradient. Fractions collected from the gradient were analysed by gel electrophoresis, followed by Western blot analysis (data not shown). Numbers of protein molecules linked per liposome were calculated by determining the amount of lipid based on DiO fluorescence and the protein concentration. To liposomes of 100±23 nm diameter an average of 220 scFv were attached, corresponding to a coupling efficiency of 15±3%.

### Binding of scFv-liposomes to ED-B positive cells *in vitro*

**Figure 1:**
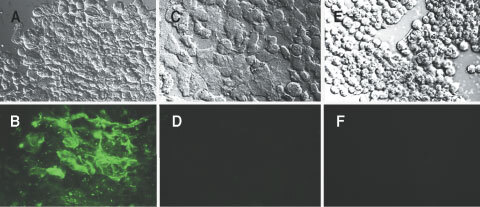
Binding of anti-ED-B scFv-liposomes to cells cultured for 48 h on collagen-I coated cover slips: (**A, C, E**) Phase contrast images of the sections shown in (**B, D, F**). ScFv-liposomes on ED-B positive Caco-2 cells (**B**) unmodified liposomes on ED-B positive Caco-2 cells (**D**) scFv-liposomes on ED-B negative Co-115 cells (**F**). Detection of specific binding was demonstrated by labelling the liposomes with the lipophilic fluorescent dye DiO. Magnification: ×400.

Caco-2 tumour cells cultured on collagen-I express ED-B Fn (Pujuguet *et al*, 1996). The cells were incubated with DiO labelled scFv-liposomes and binding was analysed by fluorescence microscopy. Fluorescence labelled scFv-liposomes bound strongly to ED-B Fn positive Caco-2 cells (Figure 1B

) but not to ED-B Fn negative Co-115 control cells (Figure 1F). Unmodified liposomes did not bind to Caco-2 cells (Figure 1D).

### Biodistribution of scFv-liposomes in tumour bearing mice

For biodistribution studies we used F9 teratocarcinoma tumours that strongly express ED-B Fn *in vivo*, are highly vascularised and are not metastasising (Neri *et al*, 1997). Organ and tumour distribution of the liposomes is shown in Table 1

**Table 1:**
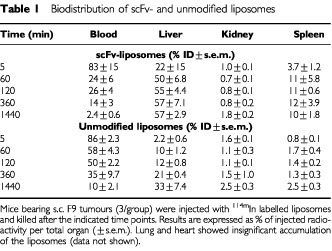
Biodistribution of scFv- and unmodified liposomes

and Figures 2

**Figure 2:**
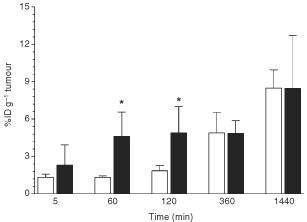
Tumour accumulation of unmodified liposomes and scFv-liposomes measured at different time points. Mice (three per group) bearing s.c. F9 tumours were injected with ^114m^In labelled liposomes and killed after different time points. Results are expressed as % injected dose of radioactivity per gram tissue (%ID g^−1^). Open bars, control liposomes; closed bars, scFv-liposomes. At 1 and 2 h after injection the difference between the two preparations was statistically significant (*P<0.05; ±s.e.m.).

and 3. Figure 2 compares the accumulation of scFv- and unmodified liposomes in tumours over a 24 h period. After 1 h the scFv-liposomes were detectable at a higher level as compared to unmodified liposomes. Unmodified liposomes reached the level of scFv-liposomes only after 6 h. The higher initial accumulation rate of the scFv-liposomes may be due to specific binding of scFv to ED-B Fn. After 6 h specific binding and penetration into the extravascular space may be saturated. Extended circulation in the blood of unmodified liposomes leads to comparable tumour accumulation.

As shown in Table 1 unmodified liposomes had the known typical distribution characteristics of small pegylated liposomes. Two hours after application 50% of the injected dose (%ID) of these liposomes were still detected in the blood, whereas only a small fraction accumulated in the tumour (Figure 2, open bars). The scFv-liposomes accumulated within 1 h at 50% ID in the liver and at 11% ID in the spleen (Table 1). The rapid accumulation in the spleen and the liver may be due to interactions between the scFv and plasma proteins that accelerate liposome uptake in the organs of the mononuclear phagocyte system (MPS, Harding *et al*, 1997). Nevertheless, the circulation time of the scFv-liposomes compared to a cytotoxic agent like free doxorubicin that is cleared from blood within minutes is still notable. The control liposomes had a half-life *t*_1/2_ of 3.7 h, whereas for the scFv-liposomes a distribution half-life *t*_1/2α_ of 52 min and an elimination half-life *t*_1/2β_ of 77 h were calculated (Figure 3A). The faster blood clearance of scFv-liposomes resulted in a 2.5-fold lower AUC_0–24 h_ than unmodified liposomes (Figure 3A) and in a higher tumour to blood ratio of 6.8 compared to 1.35 of unmodified liposomes 24 h after administration (Figure 3B).

### Therapy studies

**Figure 4:**
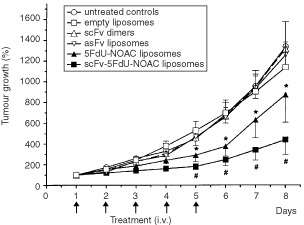
*In vivo* effects of liposome treatment on F9 tumour growth. Tumour bearing mice were treated five times every 24 h. Values represent the mean±s.e.m. of 5–10 treated mice. The tumour volumes were calculated by equation V=πab^2^/6 (a=largest tumour diameter, b=perpendicular diameter). Tumour volume values were converted in % change from baseline by equation V_t_×100/V_0_ (V_t_=volume at time t and V_0_=baseline volume). Statistical significant difference was found between empty liposomes and both 5-FdU-NOAC liposome preparations (* and # *P*<0.02; ±s.e.m.).

Different cytotoxic agents (mitoxantrone, doxorubicin, NOAC and 5-FdU-NOAC) were tested on F9 cells using a cell viability test (data not shown). The most active compound was mitoxantrone (IC_50_=2.5 μM) followed by doxorubicin (IC_50_=10 μM) and 5-FdU-NOAC (IC_50_=10 μM). We chose 5-FdU-NOAC because due to its lipophilic properties this new duplex drug can be added directly to the lipids during liposome preparation (Cattaneo-Pangrazzi *et al*, 2000), whereas loading of liposomes with mitoxantrone or doxorubicin would necessitate additional preparation steps. ScFv-5-FdU-NOAC-liposomes showed a remarkable reduction of tumour growth (Figure 4

). After 5 days of treatment tumour growth was inhibited by 62% (as calculated from the tumour diameters) compared to control mice. Untreated mice and the groups receiving control liposomes and scFv dimers showed progressive tumour growth, whereas unmodified 5-FdU-NOAC-liposomes had an intermediate anti-tumour effect. As shown in Figure 4 treatment with 5-FdU-NOAC containing liposomes was statistically significantly different from empty liposomes (*P*>0.02) on days 5 to 8. However, the statistical comparison between 5-FdU-NOAC liposomes and scFv-5-FdU-NOAC liposomes revealed no significant difference (*P*=0.14). After 8 days the tumours of untreated control mice had reached a large size requiring the sacrifice of the animals. The tumours were immediately excised and weighed. Calculated by weight a 90% reduction of tumour mass was found with the scFv-5FdU-NOAC-liposomes.

### Histology

**Figure 5:**
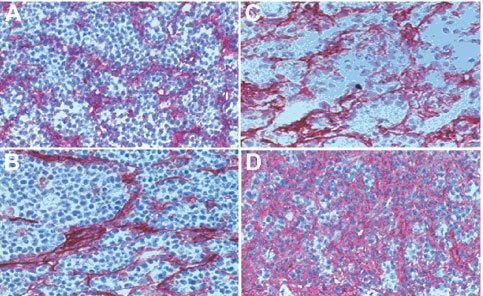
Histological analysis of F9 tumours treated with different liposome preparations. Tumours were excised 72 h after administration and sections stained with anti-ED-B scFv L19. Empty unmodified liposomes (**A**); empty scFv-liposomes (**B**); unmodified 5-FdU-NOAC-liposomes (**C**); scFv-5-FdU-NOAC-liposomes (**D**). Magnification: ×400.

To obtain histology samples from F9 tumours we injected the same liposome preparation intravenously into mice bearing tumours as used in the therapy experiments. Using an anti CD31 antibody no significant differences in endothelial cell staining were observed between all liposome preparations (data not shown). In contrast, anti-ED-B-Fn staining showed distinct differences between unloaded and 5-FdU-NOAC-liposomes (Figure 5

). Empty unmodified liposomes (Figure 5A) and empty scFv-liposomes (Figure 5B) did not change tumour tissue morphology, whereas treatment with unmodified liposomes containing the cytotoxic agent 5-FdU-NOAC (Figure 5C) resulted not only in reduction of tumour size (Figure 4), but also in a change of tissue morphology. These liposomes had remarkable cytotoxic effects on tumour cells as shown by the reduction of tumour cell number and loosened tissue architecture. At higher magnification apoptotic tumour cells were frequently seen (data not shown). Treatment of mice with specific scFv-5-FdU-NOAC-liposomes resulted in an excessive deposition of Fn in the extracellular matrix (Figure 5D). Treatment with scFv dimer did not alter tissue morphology (data not shown).

In summary, the 5-FdU-NOAC-liposome preparations had cytotoxic effects on tumour cells but not on tumour endothelial cells, whereas the ED-B Fn specific scFv-5-FdU-NOAC liposomes caused an intense deposition of Fn.

## DISCUSSION

We prepared target specific immunoliposomes by attaching functionalised scFv recognising the ED-B isoform of Fn to pegylated liposomes. We used one of the most efficient coupling methods consisting of the conjugation of thiolated proteins to liposomes containing either thiol or maleimide groups (Schwendener *et al*, 1990; Hansen *et al*, 1995). To prevent steric hindrance of antibodies that are directly linked to the lipid surface of pegylated liposomes we attached the scFv to the distal end of the PEG chains, endowing them with higher antigen accessibility and more freedom of motion. Thiol groups can easily be introduced into proteins with bispecific coupling molecules such as N-succinimidyl-S-acetylthioacetate (SATA) that interact with amino groups. However, this chemical modification may abolish the antibody binding properties as observed by modification of the anti-ED-B scFv with SATA (data not shown) and as reported by Uyama *et al* (1994). Another disadvantage of introducing active groups randomly over a protein is that the orientation of the protein molecules on the liposome surface is not controllable. Thus, by introducing reactive groups at a defined site of the protein its orientation can be predetermined. To achieve this goal we used DNA-engineering for C-terminal specific cysteine modifications of the scFv molecules. C-terminal cysteine thiols offer a wide possibility for functionalisation of the scFv molecules with the advantage of not interfering with the antigen binding domain.

With such fluorescently labelled scFv-liposomes we showed strong binding *in vitro* on ED-B positive Caco-2 cells (Figure 1). The biodistribution experiments resulted in an increased tumour uptake of scFv-liposomes up to 2 h after administration (Figures 2 and 3B). At later time points unspecific pegylated liposomes accumulated in comparable amounts (5–8 %ID g^−1^) in the tumours. This may be explained by two observations, firstly the well described high uptake of pegylated liposomes into solid tumours that is mainly a consequence of the long blood circulation times (Figure 3A). Secondly, scFv-liposomes were taken up by the liver in high amounts with 50 %ID g^−1^ after 1 h (Table 1). This reflects one of the most commonly recognised limitations for the use of antibody modified liposomes *in vivo*. The rapid clearance of scFv-liposomes from circulation is due to the opsonisation of protein coated liposomes followed by the MPS uptake (Harding *et al*, 1997). Thus, the numbers and the properties of protein molecules linked to the liposome surface are of crucial importance regarding their interactions with the MPS. As observed by others, low numbers (<10 per liposome) of scFv molecules lead to longer blood circulation times and, consequently, to higher tumour uptake (Goren *et al*, 1996; Park *et al*, 1997). As we used liposomes that were coated with up to 220 scFv molecules these rather high numbers of scFv might have contributed to the fast clearance and high uptake into the liver and the spleen. Therefore, in future experiments the number of scFv linked to a liposome will have to be optimised. Reduced liposome binding to the target might also be induced by repeated administration causing over saturation of the Fn binding domains.

In the therapy studies the mice treated with specific scFv-5-FdU-NOAC-liposomes showed a remarkable reduction in tumour size. This enhanced cytotoxic effect of scFv-5-FdU-NOAC-liposomes could be explained by the specific binding to ED-B Fn causing longer lasting liposome retention in the tumour tissue and promoting deeper penetration into the extracellular matrix. Nevertheless, together with a reduction of the number of scFv molecules per liposome, optimisation of the therapy schedule and the use of other drugs might lead to improved therapeutic modalities.

The histological examination of the F9 tumours treated with 5-FdU-NOAC containing liposomes revealed that the cytotoxic effect mainly affected tumour cells. Examination of the Fn distribution in the tumours by scFv L19 staining exposed a remarkable deposition of Fn after treatment with the scFv-5-FdU-NOAC liposomes (Figure 5D) that was possibly responsible for inhibited tumour growth.

The cytotoxic activity of non-targeted 5-FdU-NOAC-liposomes is noteworthy. 5-FdU-NOAC is a drug representative for a series of new amphiphilic/lipophilic nucleoside duplex drugs that were developed by us (Cattaneo-Pangrazzi *et al*, 2000). The chemical combination of 5-FdU (floxuridine) with NOAC that is a lipophilic derivative of cytosine arabinoside (Horber *et al*, 2000) to a new heterodinucleoside dimer represents a new class of molecules with high activity against solid tumours and excellent properties for incorporation into liposomes. Target cell specific immunoliposomes offer the possibility to use the nucleoside duplex drugs together with other cytotoxic compounds (e.g. doxorubicin, mitoxantrone) and with anti-angiogenic molecules that could concomitantly be incorporated into the liposomes.

In conclusion, our experiments are the first to our knowledge to show inhibition of tumour growth by targeting immunoliposomes to a protein that is not exclusively expressed on tumour cells but also in the stroma and on endothelial cells of the tumour vasculature. Keeping in mind that the ED-B domain of fibronectin is conserved between mouse and humans, ED-B Fn specific scFv-liposomes containing cytotoxic and/or anti-angiogenic compounds represent a promising approach for the therapeutic targeting of solid tumours in humans.
